# Measuring Protein Content in Food: An Overview of Methods

**DOI:** 10.3390/foods9101340

**Published:** 2020-09-23

**Authors:** Maria Hayes

**Affiliations:** Food BioSciences Department, Teagasc Food Research Centre, Ashtown, 15 Dublin, Ireland; Maria.Hayes@teagasc.ie; Tel.: +353-1-8059957

**Keywords:** protein, amino acid, Kjeldahl, Dumas, Lowry, Bradford, BCA, protein digestibility corrected amino acid score (PDCAAS), digestible indispensable amino acid score (DIAAS), muscle mass

## Abstract

In order to determine the quantity of protein in food, it is important to have standardized analytical methods. Several methods exist that are used in different food industries to quantify protein content, including the Kjeldahl, Lowry, Bradford and total amino acid content methods. The correct determination of the protein content of foods is important as, often, as is the case with milk, it determines the economic value of the food product and it can impact the economic feasibility of new industries for alternative protein production. This editorial provides an overview of different protein determination methods and describes their advantages and disadvantages.

The type and quality of protein that we consume is important for our general health and wellbeing. Protein in the diet is an energy provider, but also has other purposes, including the passage of bio-chemicals across cellular membranes and enzyme activity. Moreover, sufficient intake of protein in the diet is an essential nutritional factor for the prevention of diseases such as sarcopenia in an aging global population. Muscle strength and mass both decline rapidly in our 50s and a 30–50% loss of muscle mass is often observed between the ages of 40–80 years old [1]. Protein is recognized as the nutritional factor that can slow down and even prevent the loss of muscle strength and mass, but human dietary intervention studies on muscle health performed to date have largely looked at animal-sourced protein [2]. Limited information on novel proteins including terrestrial and marine plant proteins is available. In order to determine the quantity of protein in food, it is important to have standardized analytical methods. Several methods exist that are used in different food industries to quantify the protein content in foods and these include the Kjeldahl, Lowry, Bradford and total amino acid content methods. The correct determination of the protein content of foods is important as, often, as is the case with milk and wheat, it determines the economic value of the food product [3].

The nutritional quality of protein in a food product is also important and protein quality can be defined as several things, including (i) protein to support optimal growth, (ii) amino acid balance, (iii) extent of digestion and absorption of protein, or (iv) indispensable amino acids relative to amino acid requirements. Protein quality according to the Food and Agricultural Organization of the United Nations (FAO) relates to the amino acid composition of the protein source and the bioavailability (which relates to digestibility) of the protein. Several methods for measuring protein quality also exist and usually involve costly animal studies that are used to determine the biological value, digestibility and bioavailability of the protein, which are usually achieved by measuring the nitrogen remaining in the feces and urine following feed trials and the digestion of selected diets with/without protein by the animal of choice, which is usually a pig. Comparisons are then made to “gold-standard” proteins that are known to be highly digestible and bioavailable and amino acid-rich such as gelatin [4]. Measures including biological value (BV), which can be defined as the percentage of absorbed nitrogen retained in the body, and true digestibility (D), which is defined as the percentage of true nitrogen absorbed from the gut according to the Mitchell method [5], are taken into account in protein quality calculations. The nutritive value of a protein is BV X D. Several other methods and variations exist for measuring protein quality, including the protein efficiency ratio (PER), the FAO-approved Protein Digestibility corrected amino acid score (PDCAAS) method and, more recently, the FAO-approved replacement for PDCAAS, the digestible indispensable amino acid score (DIAAS) method, which is normally carried out using a rat model [6].

However, to determine protein value, it is first essential to accurately quantify the protein content in any food source or digested food and this paper will discuss the advantages and disadvantages of protein quantification methods that are routinely used in food science and by the food industry. The most frequently used methods for measuring protein content in foods include the Kjeldahl method, Dumas method, direct measurement methods using UV-spectroscopy and refractive index measurement. Each method has advantages and disadvantages. The Kjeldahl method involves the digestion of food with a strong acid so that nitrogen is released, which is then quantified using a titration technique. Protein quantity is then calculated from the nitrogen concentration of the food using a conversion factor (usually 6.25 which is equivalent to 0.16 g nitrogen per gram of protein). This is considered the standard method for protein measurement but has its disadvantages. As discussed previously by Maehre et al. [7] and others [8,9], it does not measure true protein and the conversion/correction factor of 6.25 is not suitable for all protein types and should be corrected based on the amino acid composition of the protein in question. A number of studies have identified species-specific nitrogen correction/conversion factors [7,8,9,10]. For example, a conversion factor of 5.6 is recommended for shrimp and fish, 5.4 for cereal products and 4.59 for red seaweed. However, other authors have recommended conversion factors of 4.9 for fish and 4.7 for flour [7]. The Dumas method is fast and does not use chemicals, but is costly to set up and is not very accurate as it does not measure true protein. UV spectrophotometric methods, including the Biuret, Bradford and Lowry methods are easy to use, not costly and can quantify small amounts of protein. However, they can give false positive protein readings depending on the sample preparation method used and solubility of the test sample. Direct amino acid analysis involves hydrolysis of the protein with HCL and subsequent quantification of the amino acids using HPLC. Table 1 outlines the advantages and disadvantages of different protein quantification methods.

To make direct comparisons of protein quantities determined for foods in different studies is difficult based on the number of methods that are available. The method chosen for protein quantification should be justified by the purpose of the study. Maehre and colleagues [7] assessed the protein content of a number of different foods including cod, salmon, shrimp and dulse (the red seaweed *Palmaria palmata*) and flour. Proteins were extracted using different methods and then quantified using direct amino acid analysis, the Bradford method, the modified Lowry method and the Kjeldahl method. Direct amino acid analysis was used as the reference standard in this work in accordance with the recommendations given by the Food and Agricultural Organization of the United Nations (FAO) regarding the determination of food proteins [18]. This study found that the Kjeldahl method overestimated the quantity of protein in these different foods by between 40–71%, even when the species-specific conversion/correction factor for nitrogen was used. This study and others have shown that measuring protein based on nitrogen conversion factors overestimates the protein content. This is problematic as the economic value of the food is often determined by the protein quantity. Furthermore, the manufacture of protein-blended products could be affected by the overestimation of protein in an ingredient. Moreover, the overestimation of protein content in novel and emerging alternative proteins such as seaweed, insect protein and other plant protein sources also overestimates the potential for their use and the economic feasibility and value of these new protein sources. This is a particular problem in relation to the seaweed industry. A number of research studies have reported protein contents in different red, brown and green seaweeds, with reports of up to 47% protein content based on the dry weight of the seaweed reported for the red seaweed *Porphyra* sp. previously [18]. In reality, both accessing seaweed protein and extracting it are difficult due to the presence of the seaweed cell wall and the fact that protein binds to the carbohydrate fraction and, furthermore, when the results of the protein content of certain seaweeds were determined previously using amino acid analysis and compared to the Dumas or Kjeldahl method, the actual protein content found with amino acid analysis was lower than the quantities reported using Dumas or Kjeldahl. From this perspective, the careful selection of protein determination methods is necessary, and the same methods should be compared (like for like) to ensure the correct reporting of protein quantities in foodstuffs. In addition, in accordance with the FAO, the amino acid analysis method provides the most accurate measurement of protein content in foods and should be used where possible.

## Figures and Tables

**Table 1:** Protein quantification methods—advantages and disadvantages.

Protein Quantification Method	Advantages	Disadvantages	References
Kjeldahl method—digestion of food with a strong acid so that nitrogen is released which is then quantified using a titration technique.	Considered the standard method globally and therefore easy to compare results with other laboratories	Does not measure true protein and overestimations of protein can result due to use of standard nitrogen correction factor 6.25	[7,11]
Dumas method	Fast and does not use chemicals; can measure several samples at a time	Costly to set up and is not very accurate as it does not measure true protein.	[7,12]
UV spectroscopy methods	Simple, does not require any assay agents	Highly error prone due to other compounds that absorb at the selected absorbance wavelength (280 nm)	[7,13]
Biuret methods—protein–copper chelation and secondary detection of reduced copper, includes the bicinchoninic acid (BCA) and Lowry assay methods	Less protein–protein variation than the Coomassie dye-based assays; compatible with most surfactants used for protein extraction	Incompatible with copper-reducing surfactants and reducing agents including DTT	[7,14]
Bradford Coomassie Blue assay method—protein–dye binding and direct detection of the color change	Fast, performed at room temperature, compatible with most solvents	High protein–protein variation; incompatible with detergents	[7,15]
Fluorescent dye methods—protein–dye binding and direct detection of increase in fluorescence associated with the bound dye includes the Qubit assay and EZQ ^TM^ assay	Very sensitive and uses less protein	Requires a fluorescence detector	[7,16]
Direct amino acid analysis using hydrolysis and HPLC quantification	Accurate	Initial investment in HPLC equipment required; hydrolysis step required; time consuming	[7,17]

## References

[1] Ni Lochlainn M., Bowyer R.C.E., Steves C.J. (2018). Dietary protein and muscle in aging people: The role of the gut microbiome. Nutrients.

[2] Hackney K.J., Trautman K., Johnson N., Mcgrath R., Stastny S. (2019). Protein and muscle health during aging: Benefits and concerns related to animal-based protein. Anim. Front..

[3] Miao R., Hennessy D.A. (2011). Economic Value of Information: Wheat Protein Measurement. Ph.D. Thesis.

[4] Loveday S.M. (2019). Food Proteins: Technological, Nutritional and sustainability attributes of traditional and emerging proteins. Annu. Rev. Food Sci. Technol..

[5] Mitchell H.H., Hamilton T.S., Beadles J.R.J. (1952). The relationship between the protein content of corn and the nutritional value of the protein. J. Nutr..

[6] Rutherfurd S.M., Fanning A.C., Miller B.J., Moughan P.J. (2015). Protein digestibility, corrected amino acid scores and digestible indispensable amino acid scores differentially describe protein quality in growing male rats. J. Nutr..

[7] Maehre H.K., Dalheim L., Edvinsen S.K., Elvevoll E.O., Jensen I.-J. (2018). Protein determination—Methods Matter. Foods.

[8] Harrysson H., Hayes M., Eimer F., Carlsson N.-G., Toth G.B., Undeland I. (2018). Production of protein extracts from Swedish red, green and brown seaweeds, *Porphyra umbilicalis* Kützing, *Ulva lactuca* Linnaeus, and *Saccharina latissima* (Linnaeus) J. V Lamouroux using three different methods. J. Appl. Phycol..

[9] Mariotti F., Tomé D., Miranda P.P. (2008). Converting nitrogen into protein—Beyond 6.25 and Jones’ factors. Crit. Rev. Food Sci. Nutr..

[10] Biancarosa I., Espe M., Bruckner C.G., Heesch S., Liland N., Waagbo R., Torstensen B., Lock E.J. (2017). Amino acid composition, protein content, and nitrogen to protein conversion factors of 21 seaweed species from Norwegian waters. J. Appl. Phycol..

[11] Wybraniec S., Michalowski T., Garcia Asuero A. (2013). An overview of the Kjeldahl method of nitrogen determination. Part II. Sample preparation, working scale, instrumental finish and quality control. Crit. Rev. Anal. Chem..

[12] Shea F., Watts C.E. (1939). Dumas method for organic nitrogen. Ind. Eng. Chem. Anal..

[13] Stoscheck C.M. (1990). Quantitation of Protein. Methods Enzymol..

[14] Zheng K., Wu L., He Z., Yang B. (2017). Measurement of the total protein in serum by biuret method with uncertainty evaluation. Measurement.

[15] Kruger N.J., Walker J.M. (2019). The Bradford Method for protein quantitation. The Protein Protocols Handbook.

[16] Nakayama Y., Yamaguchi H., Einaga N., Esumi M. (2016). Pitfalls of DNA quantification using DNA-binding fluorescent dyes and suggested solutions. PLoS ONE.

[17] Rutherfurd S.M. (2009). Accurate determination of the amino acid content of selected feedstuffs. Int. J. Food Sci. Nutr..

[18] FAO (2003). Food Energy—Methods of Analysis and Conversion Factors.

